# The effect of trichlormethiazide in autosomal dominant polycystic kidney disease patients receiving tolvaptan: a randomized crossover controlled trial

**DOI:** 10.1038/s41598-021-97113-w

**Published:** 2021-09-03

**Authors:** Kiyotaka Uchiyama, Chigusa Kitayama, Akane Yanai, Yoshitaka Ishibashi

**Affiliations:** 1 Division of Endocrinology, Metabolism and Nephrology Department of Internal Medicine, Keio University School of Medicine, 35 Shinanomachi, Shinjuku-ku, Tokyo, 160-8582 Japan; 2 Division of Nephrology, Japanese Red Cross Medical Center, 4-1-22 Hiroo, Shibuya-ku, Tokyo, 150-8935 Japan; 3 Department of Nephrology, Japan Community Health Care Organization (JCHO) Sendai Hospital, Miyagi, 981-8501 Japan; 4 Department of Nephrology, Tokyo Shinagawa Hospital, Tokyo, 140-8522 Japan

**Keywords:** Nephrology, Kidney diseases, Quality of life, Prognostic markers

## Abstract

The vasopressin V2 receptor antagonist tolvaptan delays the progression of autosomal dominant polycystic kidney disease (ADPKD). However, some patients discontinue tolvaptan because of severe adverse aquaretic events. This open-label, randomized, controlled, counterbalanced, crossover trial investigated the effects of trichlormethiazide, a thiazide diuretic, in patients with ADPKD receiving tolvaptan (n = 10) who randomly received antihypertensive therapy with or without trichlormethiazide for 12 weeks. The primary and secondary outcomes included amount and osmolarity of 24-h urine and health-related quality-of-life (HRQOL) parameters assessed by the Kidney Disease Quality of Life-Short Form questionnaire, renal function slope, and plasma/urinary biomarkers associated with disease progression. There was a significant reduction in urine volume (3348 ± 584 vs. 4255 ± 739 mL; *P* < 0.001) and a significant increase in urinary osmolarity (182.5 ± 38.1 vs. 141.5 ± 38.1 mOsm; *P* = 0.001) in patients treated with trichlormethiazide. Moreover, trichlormethiazide improved the following HRQOL subscales: effects of kidney disease, sleep, emotional role functioning, social functioning, and role/social component summary. No significant differences were noted in renal function slope or plasma/urinary biomarkers between patients treated with and without trichlormethiazide. In patients with ADPKD treated with tolvaptan, trichlormethiazide may improve tolvaptan tolerability and HRQOL parameters.

## Introduction

Autosomal dominant polycystic kidney disease (ADPKD) is the most common genetic renal disorder and one of the leading causes of end-stage kidney disease^1^. The vasopressin V2 receptor antagonist tolvaptan has been recently shown to delay the increase in total kidney volume (TKV) as well as decline in kidney functions compared with placebo in a randomized controlled trial (RCT) of patients with ADPKD with near-normal kidney functions and late-stage ADPKD^2,3^. However, tolvaptan usage has been implicated in polyuria, with an increase in urinary free water excretion related to the drug’s mechanism of action, and it has been established as a diuretic for treatment of volume overload in heart failure in Japan at the dose of 7.5–15 mg^4,5^. However, at least 8.3% of those who received tolvaptan at 60–120 mg dosage unfortunately discontinued the trial when they developed aquaresis-related symptoms^2^.

Thiazide diuretics have long been known to function paradoxically and improve polyuria in patients with nephrogenic diabetes insipidus in whom the vasopressin V2 receptor function is absent^6^, although the underlying mechanism remains unestablished. Therefore, we hypothesized that thiazide diuretics may reduce the urinary volume in patients with ADPKD receiving high-dose tolvaptan, in whom the vasopressin V2 receptor is pharmacologically blocked. In fact, a recent case indicated that the addition of hydrochlorothiazide could reduce polyuria with an increase in urinary osmolarity (Uosm), which may have improved tolvaptan tolerability in patients with ADPKD^7^. However, a clinical trial is necessary to verify whether the effect of co-prescription (thiazide + tolvaptan) is scientifically valid.

Therefore, we designed an open-label, pilot, randomized, controlled, crossover trial involving patients with ADPKD receiving tolvaptan to primarily clarify the effect of trichlormethiazide, the most popular thiazide agent in Japan, in improving tolvaptan tolerability as well as to assess its impact on health-related quality-of-life (HRQOL) parameters of patients as one of the secondary endpoints. In addition, as the case report indicated that thiazide deteriorates the effect of tolvaptan on the rate of ADPKD progression, we evaluated the short-term effects of trichlormethiazide on renal function and plasma/urinary biomarkers.

## Results

### Baseline characteristics

**Figure 1:**
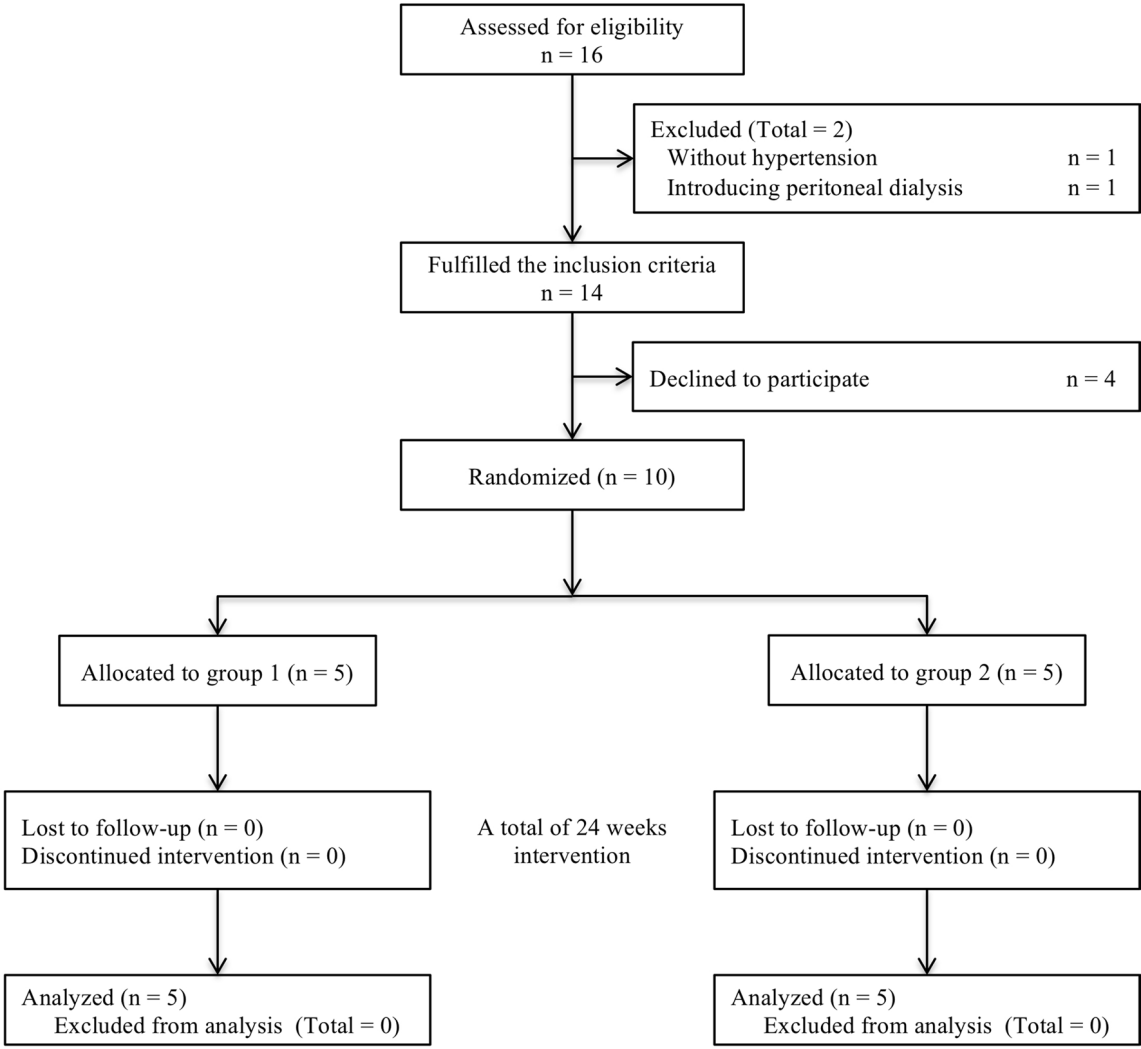
CONSORT flow diagram of patients through the various phases of the trial process.

Among the 16 patients with ADPKD receiving tolvaptan who were assessed for their eligibility, 14 fulfilled the inclusion criteria and pre-targeted 10 patients gave consent for study participation (see Fig. 1). We randomized 10 participants in a crossover design to first prescribe antihypertensive treatment either with trichlormethiazide (group 1, n = 5) or without any thiazide diuretics (group 2, n = 5), and all participants completed the study period of intervention with no missing data. The baseline characteristics of all participants are depicted in Table 1, and they were not significantly different between the groups. Notably, young age and high body mass index, suggesting a large muscle mass, might lead to lower creatinine (eGFR_Cr)_ value estimation compared with cystatin C (eGFR_Cys_) value estimation, particularly in patients in Group 2; however, it was difficult to exactly determine the reason for this discordance between eGFR_Cr_ and eGFR_Cys_. In addition, paired *t*-test for values of the first visit of each trial demonstrated that the order effect was statistically avoided for most of the anthropometric and biochemical data measured (see Table S1).

**Table 1:** Demographic, clinical, and biochemical data of the study groups.

Variables	All (n = 10)	Group 1 (n = 5)*	Group 2 (n = 5) †	*P* value
Age (year)	49 (44–71)	51 (47–70)	44 (41–72)	0.69
Male/female (%male)	4/6 (40/60%)	2/3 (40/60%)	2/3 (40/60%)	1
Smoking (%)	0 (0%)	0 (0%)	0 (0%)	1
Family history of ADPKD	8 (80%)	4 (80%)	4 (80%)	1
Tolvaptan (mg)	120 (90–120)	120 (90–120)	120 (60–120)	0.72
Duration of tolvaptan treatment (months)	43 (13–50)	28 (8–44)	51 (42–53)	0.22
BMI (kg/m^2^)	24.1 (22.6–27.5)	22.8 (22.0–26.3)	24.1 (24.0–36.4)	0.55
GNRI	111.3 ± 11.7	105.0 ± 5.3	117.5 ± 13.4	0.09
Systolic BP (mmHg)	129.8 ± 11.7	135.0 ± 13.7	124.6 ± 7.0	0.17
Diastolic BP (mmHg)	81.2 ± 12.4	85.6 ± 12.1	76.8 ± 12.4	0.29
Mean BP (mmHg)	97.4 ± 11.7	102.1 ± 12.3	92.7 ± 10.1	0.23
**Complications of ADPKD**				
Liver cyst	6 (60%)	3 (60%)	3 (60%)	1
Brain aneurysm	2 (20%)	1 (20%)	1 (20%)	1
Valvular disease	1 (10%)	1 (20%)	0 (0%)	1
**Past medical history**				
Cyst infection	3 (30%)	1 (20%)	2 (40%)	1
Cyst hemorrhage	0 (0%)	0 (0%)	0 (0%)	1
CCVD (%)	0 (0%)	0 (0%)	0 (0%)	1
**Kidney function**				
eGFR_Cre_ (mL/min/1.73 m^2^)	39.3 ± 20.7	29.1 ± 9.6	49.5 ± 24.7	0.12
eGFR_Cys_ (mL/min/1.73 m^2^)	50.4 ± 33.6	35.5 ± 14.7	65.3 ± 42.0	0.17
UPCR (g/gCre)	0.15 (0.10–0.30)	0.10 (0.10–0.30)	0.20 (0.10–0.40)	0.5
Urinary osmolarity (mOsm)	136.4 ± 39.8	134.4 ± 31.6	138.4 ± 50.5	0.88
**Serum biochemical analyses**				
AST (IU/L)	16.7 ± 3.2	15.6 ± 1.5	17.8 ± 4.3	0.31
ALT (IU/L)	13.9 ± 5.9	13.2 ± 7.1	14.6 ± 5.1	0.73
γ-GTP (IU/L)	22 (18–28)	22 (16–23)	22 (18–29)	0.75
Sodium (mEq/L)	140.8 ± 2.8	140.4 ± 2.5	141.2 ± 3.3	0.68
Potassium (mEq/L)	4.46 ± 0.54	4.60 ± 0.60	4.32 ± 0.50	0.45
Chloride (mEq/L)	106.0 ± 3.5	107.0 ± 3.2	105.0 ± 3.8	0.4
Albumin (g/L)	4.10 ± 0.29	3.96 ± 0.15	4.24 ± 0.34	0.13
Fasting blood sugar (mg/dL)	110.8 ± 21.5	104.0 ± 10.7	117.6 ± 28.5	0.35
LDL cholesterol (mg/dL)	111.4 ± 28.2	103.8 ± 22.5	119.0 ± 33.7	0.43
HDL cholesterol (mg/dL)	53.0 ± 9.4	51.6 ± 9.7	54.4 ± 10.0	0.67
Triglyceride (mg/dL)	106.0 ± 3.5	123.6 ± 39.1	103.0 ± 28.0	0.37
CRP (mg/L)	0.12 ± 0.09	0.11 ± 0.06	0.12 ± 0.12	0.85
BNP (pg/mL)	20.0 (8.5–25.4)	20.5 (13.9–25.4)	16.4 (8.1–42.7)	0.89
Hemoglobin (g/dL)	12.6 ± 1.2	12.3 ± 0.6	13.0 ± 1.6	0.36
Uric acid (mg/dL)	6.35 ± 1.44	6.32 ± 0.84	6.38 ± 1.98	0.95
TKV (mL)	1458 (1159–1905)	1462 (1454–1987)	1359 (1017–1878)	0.69
htTKV (mL/m)	876 (693–1165)	881 (871–1183)	871 (634–1111)	0.84

*ADPKD* autosomal dominant polycystic kidney disease, *BMI* body mass index, *GNRI* geriatric nutritional risk index, *BP* blood pressure, *CCVD* cerebrovascular/cardiovascular disease, *eGFR* estimated glomerular filtration rate, *Cr* creatinine, *Cys* cystatin C, *UPCR* urine protein-to-creatinine ratio, *AST* aspartate aminotransferase, *ALT* alanine aminotransferase, *γ-GTP* γ-glutamyl transpeptidase, *LDL* low density lipoprotein, *HDL* high density lipoprotein, *CRP* C-reactive protein, *BNP* brain natriuretic peptide, *TKV* total kidney volume, *htTKV* height-adjusted total kidney volume.

*Group 1 was initiated on antihypertensive treatment with trichlormethiazide. †Group 2 was initiated on antihypertensive treatment without trichlormethiazide.

### Effect of trichlormethiazide on urinary volume and osmolarity

The details of tolvaptan dose and the antihypertensive treatment undertaken in the study are stated in Table S2. A constant tolvaptan dose was maintained in all participants throughout the study period. One patient (#6) received a calcium channel blocker (CCB) but not a renin–angiotensin system (RAS) inhibitor at baseline due to a history of an allergic reaction to this drug type. To control blood pressure (BP), the number and/or the antihypertensive agent dosage were decreased in all but three patients (#6, 7, and 10), and the RAS inhibitor dosage was reduced in five patients (#1, 3, 5, 8, and 9) on switching from treatment with trichlormethiazide to that without trichlormethiazide.

**Figure 2:**
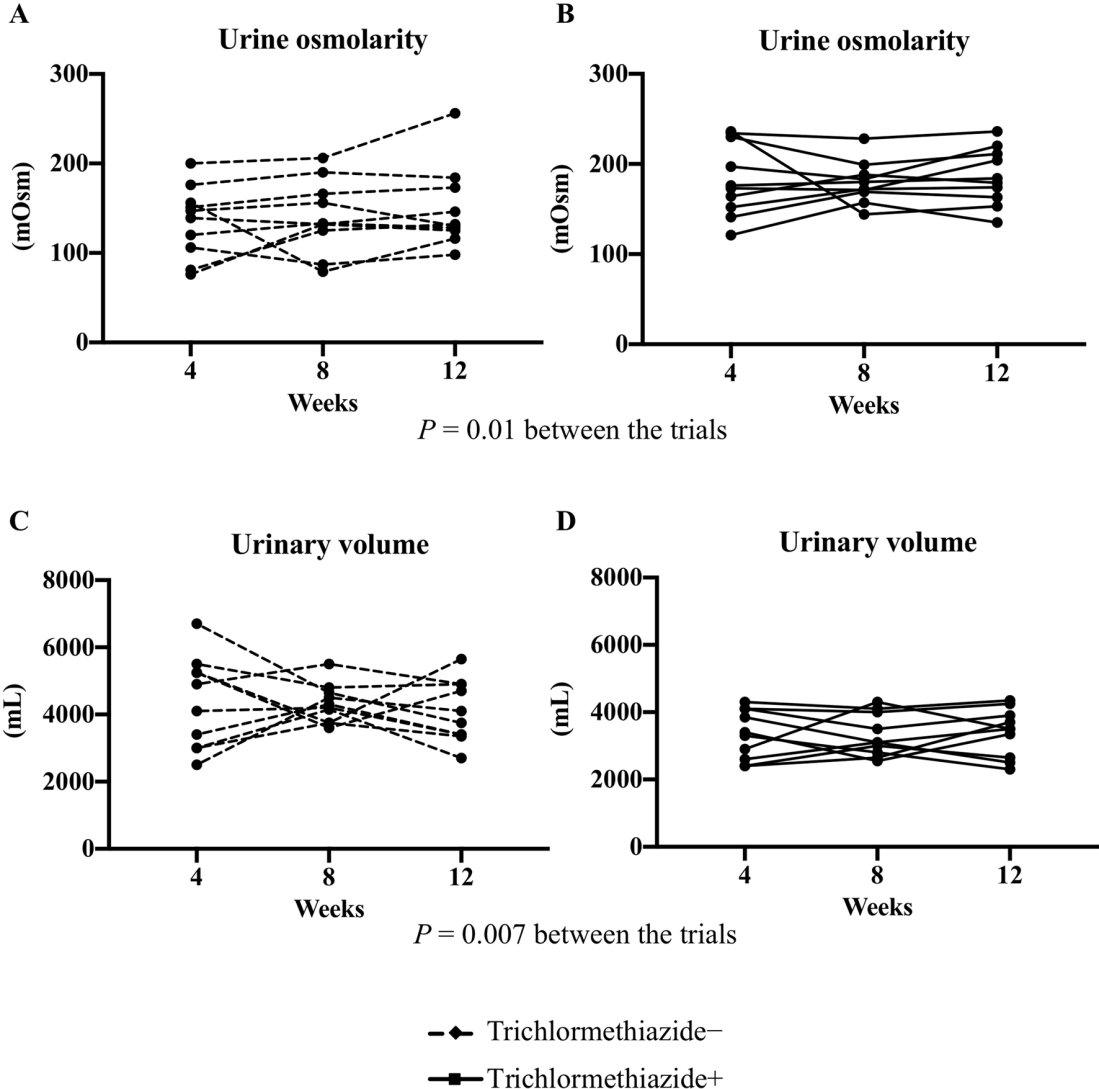
Mean change in the values of urinary osmolarity (**A**) without and (**B**) with trichlormethiazide treatment and urinary volume (**C**) without and (**D**) with trichlormethiazide treatment at 4, 8, and 12 weeks of each trial.

The mean urinary volume was significantly lower during antihypertensive treatment with trichlormethiazide than that during antihypertensive treatment without trichlormethiazide (3348 ± 584 vs. 4255 ± 739 mL; *P* < 0.001), with a significant increase in the mean Uosm of 24-h urine (182.5 ± 38.1 vs. 141.5 ± 38.1 mOsm; *P* < 0.001) (see Table 2). The mean Uosm of fasting morning spot urine sample was also significantly higher during antihypertensive treatment with trichlormethiazide than that during antihypertensive treatment without trichlormethiazide (215.5 ± 30.5 vs. 157.8 ± 47.8 mOsm; *P* = 0.004). In repeated-measured analysis of variance (ANOVA), significant differences were recorded between the trials without and with trichlormethiazide for both Uosm of 24-h urine and urinary volume (*P* = 0.01 and 0.007, respectively), albeit no significant differences were noted within the trials or within trials × time interactions for both Uosm and urinary volume (*P* = 0.41 and 0.73 [for Uosm]; *P* = 0.88 and 0.67 [for urinary volume], respectively) (Fig. 2).

**Table 2:** Effect of trichlormethiazide on urinary volume and osmolarity.

Variables	Trichlormethiazide−	Trichlormethiazide+	*P *value
Urinary volume (mL/day)	4255 ± 739	3348 ± 584	< 0.001
Urinary osmolarity (mOsm)	141.5 ± 38.1	182.5 ± 27.4	< 0.001

For these variables, the mean values at 4, 8, and 12 weeks of each trial were calculated.

The subgroup analysis to determine the potential differential effect of trichlormethiazide treatment on mean urinary volume and Uosm revealed that these parameters did not exhibit statistically significant interactions with the median duration of previous tolvaptan treatment (*P* = 0.93 and 0.98, respectively), the change in antihypertensive agent dosage during the trial (*P* = 0.24 and 0.28, respectively), and the change in RAS inhibitor dosage during the trial (*P* = 0.93 and 0.29, respectively) between the treatment with and without trichlormethiazide (Table S3).

The 24-h urinary creatinine excretion was comparable between the groups treated with and without trichlormethiazide (1.10 ± 0.22 vs. 1.10 ± 0.22 g/day, *P* = 0.85), confirming the accuracy of 24-h urinary collection during both trial phases.

### Effect of trichlormethiazide on HRQOL

The subscale of the effects of kidney disease, which represent the impact of kidney disease on patients’ daily lives, was significantly higher, whereas that of sleep was higher with borderline significance during antihypertensive treatment with trichlormethiazide than that without trichlormethiazide (*P* = 0.02 and 0.07, respectively) among the subscales of Kidney Disease Quality of Life (KDQOL), kidney disease-specific measure of HRQOL (see Table 3). Among the subscales of Short Form (SF)-36, emotional role functioning, social functioning, and role/social component summary were significantly higher during antihypertensive treatment with trichlormethiazide than that without trichlormethiazide (*P* = 0.02, 0.02, and 0.04, respectively). The number of nocturnal voids was significantly lower in antihypertensive treatment with trichlormethiazide than in that without trichlormethiazide (1.7 ± 1.2 vs. 2.7 ± 1.5 times, *P* < 0.001).

**Table 3:** Effect of trichlormethiazide on health-related quality of life.

Variables	Trichlormethiazide−	Trichlormethiazide+	*P *value
**KDQOL**			
Symptoms/problems	79.4 ± 16.6	86.4 ± 10.3	0.11
Effects of kidney disease	83.1 ± 15.9	95.0 ± 5.6	0.02
Burden of kidney disease	60.6 ± 21.1	67.5 ± 23.5	0.28
Work status	95.0 ± 15.8	95.0 ± 15.8	1
Cognitive function	89.3 ± 13.0	86.7 ± 25.3	0.61
Quality of social interaction	85.3 ± 19.6	84.0 ± 25.0	0.78
Sleep	57.8 ± 22.1	70.0 ± 16.5	0.07
Social support	88.3 ± 27.3	84.2 ± 12.7	0.64
Overall health rating	62.0 ± 18.7	67.0 ± 18.9	0.27
KDCS	79.9 ± 12.1	83.6 ± 10.7	0.19
**SF-36**			
Physical functioning	87.5 ± 19.0	90.5 ± 8.6	0.43
Physical role functioning	83.8 ± 18.2	92.5 ± 11.7	0.21
Body pain	67.9 ± 24.7	69.6 ± 27.9	0.87
General health	48.2 ± 16.4	55.6 ± 16.4	0.2
Vitality	63.1 ± 12.3	70.0 ± 15.3	0.13
Social functioning	86.3 ± 17.1	95.0 ± 8.7	0.02
Emotional role functioning	84.2 ± 16.4	95.8 ± 9.0	0.02
Mental health	75.5 ± 18.6	82.5 ± 14.4	0.23
PCS	44.2 ± 11.3	44.7 ± 8.4	0.88
MCS	49.3 ± 6.5	51.7 ± 8.7	0.28
RCS	51.9 ± 9.9	57.3 ± 5.8	0.04

*KDQOL* Kidney Disease Quality of Life, *KDCS* kidney disease component summary, *SF-36* Medical Outcomes Study 36-Item Short Form Health Survey MOS, *PCS* physical component summary, *MCS* mental component summary, *RCS* role/social component summary.

### Effect of trichlormethiazide on other ADPKD-related parameters

The slopes of both estimated glomerular filtration rates assessed by eGFR_Cr_ and eGFR_Cys_ were not significantly different between antihypertensive treatment with and without trichlormethiazide (0.73 ± 1.49 vs. − 0.29 ± 1.86 mL/min/1.73 m^2^/month, *P* = 0.28 and 0.68 ± 1.88 vs. − 0.11 ± 2.97 mL/min/1.73 m^2^/month, *P* = 0.59) (Table 4). The subgroup analysis to determine the potential differential effect of trichlormethiazide treatment on eGFR_Cr_ and eGFR_Cys_ slopes, indicated that these parameters did not exhibit statistically significant interactions with the median duration of previous tolvaptan treatment (*P* = 0.62 and 0.58, respectively), the change in antihypertensive agent dosage (*P* = 0.13 and 0.46, respectively), and the change in RAS inhibitor dosage (*P* = 0.52 and 0.59, respectively) between the treatment with and without trichlormethiazide (Table S4).

**Table 4:** Effect of trichlormethiazide on renal outcomes.

Variables	Trichlormethiazide−	Trichlormethiazide+	*P*value
**Renal function**			
eGFR_Cre_ slope (mL/min/1.73 m^2^/month)	− 0.29 ± 1.86	0.73 ± 1.49	0.28
eGFR_Cys_ slope (mL/min/1.73 m^2^/month)	− 0.11 ± 2.97	0.68 ± 1.88	0.59
**Urinary biomarkers**			
ACR (mg/gCr)	72.6 ± 76.1	68.0 ± 94.5	0.76
L-FABP (μg/gCr)	10.3 ± 10.8	13.1 ± 21.0	0.48
**ADPKD-related parameters**			
Plasma copeptin (pg/mL)	88.7 ± 43.2	85.4 ± 47.8	0.75
Plasma vasopressin (pg/mL)	2.33 ± 1.18	1.94 ± 0.93	0.18

For urinary biomarkers, the mean values at 4, 8, and 12 weeks of each trial were calculated.

*eGFR* estimated glomerular filtration rate, *Cre* creatinine, *Cys* cystatin C, *ACR* albumin-to-creatinine ratio, *L-FABP* liver-type fatty acid-binding protein, *ADPKD* autosomal dominant polycystic kidney disease.

In addition, the mean urinary liver-type fatty acid-binding protein (L-FABP) levels and albumin-to-creatinine ratio (ACR) of each trial, which serve as biomarkers of chronic kidney disease (CKD) progression, were not significantly different between each treatment (13.1 ± 21.0 vs. 10.3 ± 10.8 μg/gCr, *P* = 0.48; and 68.0 ± 94.5 vs. 72.6 ± 76.1 mg/gCr, *P* = 0.76). As a biomarker related to ADPKD progression, plasma vasopressin at the last visit (12 weeks) of each trial did not show any statistically significant difference between antihypertensive treatment with and without trichlormethiazide (1.94 ± 0.93 vs. 2.33 ± 1.18 pg/mL, *P* = 0.18). Moreover, no statistical difference was noted in plasma copeptin between the two treatments (85.4 ± 47.8 vs. 88.7 ± 43.2 pg/mL, *P* = 0.75).

### Effect of trichlormethiazide on electrolytes, uric acid, osmolar excretion, and blood pressure

The mean level of serum potassium was significantly lower in the group receiving trichlormethiazide versus that not receiving it (4.13 ± 0.61 vs. 4.45 ± 0.44 mEq/L, *P* = 0.005), whereas the mean value of serum sodium in each treatment was not significantly different between the respective groups (139.8 ± 2.4 vs. 140.7 ± 1.8 mEq/L, *P* = 0.09) (Table 5). One patient developed hypokalemia with a minimum serum potassium level of 3.0 mEq/L at the last trial follow-up visit during treatment with trichlormethiazide. On the other hand, the urinary excretion of either sodium or potassium was not significantly different between the groups (*P* = 0.88 and 0.96, respectively), which suggests that a steady state, in which the sodium and potassium intake and excretion were nearly the same, was established in the group receiving trichlormethiazide treatment.

**Table 5:** Effect of trichlormethiazide on serum and urinary electrolytes; serum uric acid, urinary urea nitrogen, and osmolar excretion; and blood pressure.

Variables	Trichlormethiazide−	Trichlormethiazide+	*P *value
**Electrolytes**			
Serum Na (mEq/L)	140.7 ± 1.8	139.8 ± 2.4	0.09
Serum K (mEq/L)	4.45 ± 0.44	4.13 ± 0.61	0.005
Urinary Na excretion (mEq/day)	116.6 ± 25.2	117.4 ± 29.5	0.88
Urinary K excretion (mEq/day)	43.9 ± 9.0	45.7 ± 13.7	0.56
Serum uric acid (mg/dL)	5.93 ± 1.20	7.10 ± 1.46	< 0.001
Urinary urea nitrogen excretion (mmol/day)	232.1 ± 51.5	252.4 ± 36.2	0.28
Urinary osmolar excretion (mOsm/day)	584.3 ± 134.0	605.1 ± 113.2	0.48
**Blood pressure**			
Systolic BP (mmHg)	129.0 ± 8.8	124.9 ± 9.2	0.13
Diastolic BP (mmHg)	81.9 ± 6.5	80.9 ± 6.3	0.63
Mean BP (mmHg)	97.6 ± 6.4	95.6 ± 5.9	0.34

For these variables, the mean values at 4, 8, and 12 weeks of each trial were calculated.

*Na* sodium, *K* potassium, *BP* blood pressure.

The mean serum uric acid level was significantly higher in the group treated with trichlormethiazide than in the group not treated with trichlormethiazide (7.10 ± 1.46 vs. 5.93 ± 1.20 mg/dL, *P* < 0.001). The mean 24-h urinary urea nitrogen and osmolar excretions were comparable between the groups treated with and without trichlormethiazide (252.4 ± 36.2 vs. 232.1 ± 51.5 mmol/day, *P* = 0.28 and 605.1 ± 113.2 vs. 584.3 ± 134.0 mOsm/day, *P* = 0.48, respectively).

Finally, systolic, diastolic, and mean BP values were not significantly different between the groups treated with and without trichlormethiazide (*P* = 0.13, 0.63, and 0.34, respectively), which minimized the effect of BP on the differences in other parameters, including the renal outcomes, between the trials. Additionally, none of the patients fulfilled the treatment discontinuation BP criteria of > 180/110 or < 110/60 mmHg during the study period; therefore, all patients completed the trial.

## Discussion

The present study is the first pilot RCT to describe the effect of trichlormethiazide, a thiazide diuretic, on reducing urinary volume while increasing Uosm in patients with ADPKD receiving high-dose tolvaptan. In addition, we found that the use of trichlormethiazide improved several subscales of HRQOL. Although a previous case report suggested that hydrochlorothiazide accelerates the rate of eGFR decline in association with increased serum copeptin^7^, we did not detect any signs of CKD or ADPKD progression with the use of trichlormethiazide in the present study.

Thiazide and thiazide-like diuretics primarily inhibit NaCl cotransport in the distal convoluted tubule, and they have been a mainstay in the therapy of primary hypertension^8,9^. The hypotensive effect of thiazide is initially mediated by a modest reduction in the plasma volume and cardiac output^10^; however, at the same time, the drug has a paradoxical antidiuretic effect on polyuria in nephrogenic diabetes insipidus^6,11^. This effect is presumably mediated via a hypovolemia-induced increase in sodium and water reabsorption in the proximal tubules, which leads to diminished water delivery to the vasopressin-sensitive site in the collecting ducts and thereby a decrease in urine output. Aquaretic events are the most common adverse events due to which tolvaptan usage is discontinued in patients with ADPKD^2,3^. Salt reduction may not only slow the progression of ADPKD but also reduce urine output with water intake and should be recommended first and foremost^12–14^. Additionally, the addition of trichlormethiazide to high-dose tolvaptan is expected to attenuate aquaretic events and to prevent the discontinuation of treatment with tolvaptan. Moreover, even among the patients with ADPKD who tolerate tolvaptan, the addition of trichlormethiazide to the treatment improved several HRQOL subscales, including the effect of kidney disease, sleep, and several social scores, in association with a decrease in the number of nocturnal voids and the expected decrease in the number of daily voids, which allowed better participation in daily activities. Although the HRQOL of patients with ADPKD receiving tolvaptan remains unclear, the HRQOL of non-dialysis patients with ADPKD has been reported to be impaired compared with that of the general population^15^. A small-scale study suggested that tolvaptan did not significantly affect HRQOL in only patients with ADPKD who tolerated the treatment^16^. Patients with ADPKD ideally take tolvaptan semi-permanently until the development of end-stage renal disease. Therefore, HRQOL of patients with ADPKD during tolvaptan treatment is believed to be one of the important outcomes along with other clinical outcomes, such as CKD progression and all-cause mortality.

In a study assessing the determinants of urine volume in patients with ADPKD on tolvaptan, Kramers et al. reported that the 24-h urinary volume was higher than that observed in the present study (5930 ± 1790 vs. 4255 ± 739 mL)^14^. At the same time, osmolar intake (including sodium and urea nitrogen) was shown to be the primary determinant of urine volume during treatment with tolvaptan. The 24-h urinary excretions of sodium, urea nitrogen, and osmolytes in the present study were approximately 30% lower than those reported by Kramers et al. (117.4 ± 29.5 vs. 149 ± 53 mEq/day, 252.4 ± 36.2 vs. 378 ± 109 mmol/day, and 605.1 ± 113.2 vs. 858 ± 231 mOsm/day, respectively). Therefore, it was expected that the urinary volume in the present study was approximately 30% lower than that reported by Kramers et al.

The literature demonstrates that both the circulating and intrarenal RAS activities are increased in patients with ADPKD^17^. In addition, fluid loss as a result of thiazide diuretics as well as dietary sodium restriction may lead to the activation of RAS^18^, albeit also explains the synergistic response between diuretics and a RAS inhibitor^19,20^. In fact, in the present study, trichlormethiazide along with a decreased dose of RAS inhibitor showed a comparable antihypertensive effect to that of antihypertensive treatment without trichlormethiazide, including RAS inhibitors alone or in combination with CCB, suggesting the synergistic effect of co-prescription (RAS inhibitor + thiazide).

Albuminuria is a risk factor for not only CKD progression, cardiovascular mortality, and all-cause mortality^21,22^ but also for a decline in kidney functions and an increase in TKV in patients with ADPKD^23,24^. Meanwhile, the urinary excretion of L-FABP, which is expressed in the human proximal tubules and reflects various stresses on proximal tubules, predicts the progression of non-diabetic and diabetic CKD^25–27^. Although the association of urinary L-FABP with the progression of ADPKD remains unexplained, the increase in the urinary L-FABP level in patients with ADPKD, especially in those with decreased renal function, in comparison with that in healthy individuals has been reported, suggesting the association of urinary L-FABP level with ADPKD progression^28^. In the present study, these biomarkers were comparable between the trials without and with trichlormethiazide, in addition to the similarities in eGFR slopes. Despite these findings, the small sample size and the short duration of the present trial made it impossible to reject the hypothesis that the concomitant use of a thiazide diuretic and tolvaptan in patients with ADPKD could accelerate the rate of eGFR decline, a theory that was proposed in a case report^7^.

Moreover, we focused on and measured the biomarkers specific to ADPKD progression, namely plasma vasopressin and copeptin. While the plasma vasopressin is difficult to measure, copeptin, which consists of the C-terminal portion of the vasopressin precursor, is easy to measure and a stable substitute for the circulating vasopressin^29,30^. In the TEMPO 3:4 trial, the baseline plasma copeptin level independently predicted the eGFR decline and kidney growth over a 3-year period in placebo-treated participants with ADPKD^31^. In contrast, the plasma copeptin concentration significantly increased after 3 weeks of treatment with tolvaptan^32^, and a larger percent increase in copeptin concentration from the baseline value to that at week 3 exhibited less eGFR decline and kidney growth after 3 years^31^. Thus, while copeptin is a promising biomarker for the prediction of outcome in patients with ADPKD without tolvaptan treatment, increased plasma copeptin in patients with ADPKD receiving tolvaptan should be interpreted with caution. Although an increased level of plasma copeptin has been reported in a tolvaptan–thiazide combination therapy in a previous case report, it does not necessarily indicate any direct association with the decline in kidney function^7^. Consequently, we could not detect any statistical differences in any of these ADPKD-related biomarkers between the groups without and with trichlormethiazide. An experiment using a PCK rat strain identified reduced polyuria induced by tolvaptan without ameliorating its beneficial effects on PKD^33^.

The present study has several limitations. First and most importantly, this trial was a pilot study that included a small number of participants who were monitored for a short follow-up duration. In particular, because we could not deny the possibility that thiazide diuretics adversely affect renal function, a finding of a case report, the sample size was set at a minimum value^7^. The effect of trichlormethiazide combined with tolvaptan in reducing urinary volume while increasing Uosm was definitive despite the small size of the trial; however, we cannot eliminate the possibility that trichlormethiazide may exert a negative effect on declining kidney function with only 3 months of follow-up. Therefore, future trials with a larger sample size and longer follow-up periods are warranted for a more definitive demonstration of the positive or negative impacts of trichlormethiazide on renal outcomes in patients with ADPKD receiving tolvaptan. Nevertheless, a recent paper has reported that thiazide diuretics do not have a detrimental effect on the rate of disease progression in patients with ADPKD not receiving tolvaptan and has suggest that these drugs can be prescribed as second-line antihypertensives^34^. Second, owing to the design of this open-label trial that did not blind the participants and doctors, the effect of several biases, including the placebo effect and observational bias, were undeniable, although urinary volume and urinary osmolarity were relatively objective parameters. HRQOL parameters are subjective and susceptible to bias; however, we also believe that these parameter-related biases could be reduced by not informing patients about the details of how trichlormethiazide could impact their daily lives. Moreover, the crossover design used in this trial could have also potentially added several limitations, including the order effect and the carry-over effect, although the order effect is statistically avoided as a result of comparison between the first visit of the trials without or with trichlormethiazide. Future large-scale RCTs with double-blinded and placebo-controlled designs are warranted. Finally, we did not examine the effect of the intervention on TKV, which is one of the most important outcomes in patients with ADPKD, because we considered that the 3-month period of the trials was too short for detecting differences in cyst growth between the trials. We believe that future trials with longer-term follow-up should include TKV growth as one of the study outcomes.

In conclusion, this is the first RCT to indicate the beneficial effects of trichlormethiazide on improving the tolerability to tolvaptan and HRQOL without affecting the renal outcomes in patients with ADPKD. However, our results should be interpreted cautiously with due consideration to the several study limitations. Therefore, the abovementioned effectiveness of trichlormethiazide should probably be first considered for cases that are extremely sensitive to aquaretic symptoms and are at a high risk of withdrawal from tolvaptan treatment^35^.

## Methods

### Study population

Outpatients with ADPKD at the Nephrology Department of the Japanese Red Cross Medical Center in Tokyo, Japan were included in this RCT from July 2019. The trial was completed in September 2020. The criteria for trial participation included stable patients aged > 20 years diagnosed with ADPKD based on the Japan-specific diagnostic criteria proposed by the Ministry of Health, Labor and Welfare^36^ and receiving high-dose tolvaptan (> 60 mg/day) whose disease was complicated with hypertension (home BP > 135/85 mmHg, office BP > 140/90 mmHg) or those currently using antihypertensive drugs^37^, considering that trichlormethiazide is primarily used to treat hypertension. The exclusion criteria included uncontrolled BP (> 180/110 mmHg or < 100/60 mmHg); apparent electrolyte disturbance that may worsen by trichlormethiazide use (serum sodium level < 135 mEq/L or serum potassium level < 3.5 mEq/L); symptomatic coronary artery disease or cerebrovascular disease within 3 months before trial recruitment; current uncontrolled heart failure (New York Heart Association classes III and IV); symptomatic and fatal arrhythmia; and significant valvular heart disease. Patients allergic to any of the thiazide drugs were also excluded from the trial.

### Study design and randomization

The study protocol was reviewed and approved by the ethics committee of the Japanese Red Cross Medical Center (approval number: 979) and adhered to the principles of the Declaration of Helsinki. Written informed consent was obtained from all participants. The study was registered in a public trial registry (UMIN-CTR number: UMIN000037550; 31/07/2019).

**Figure 3:**
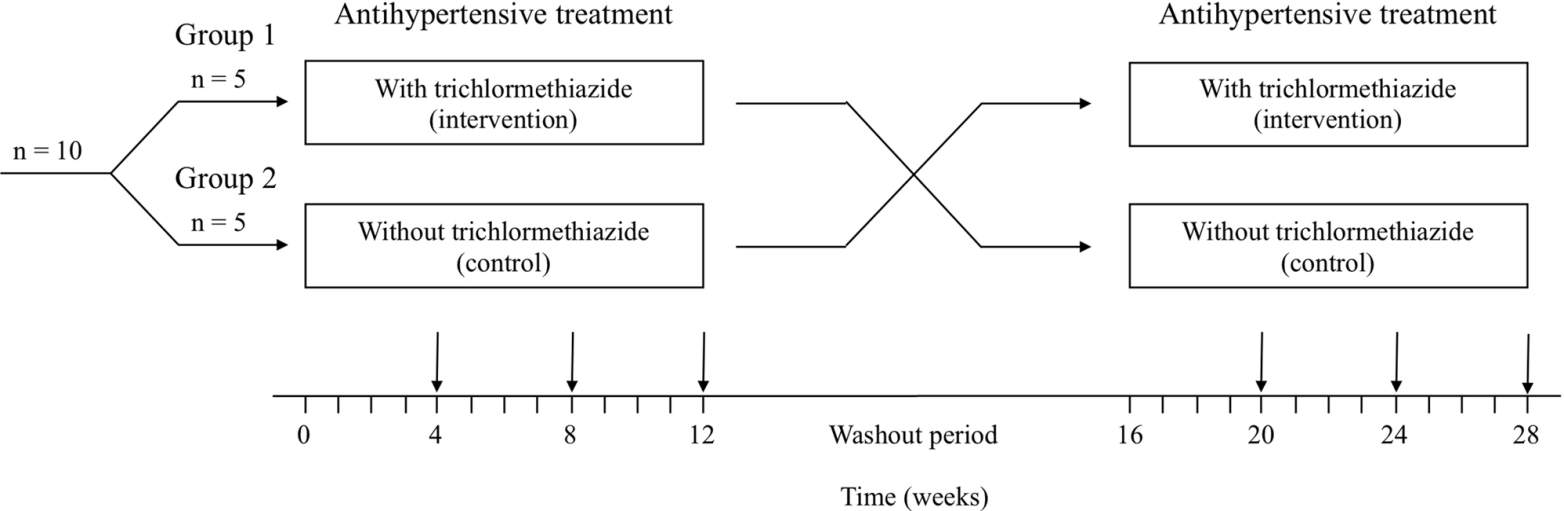
Crossover flow chart of the trial process.

After baseline assessment, an individual not associated with this study performed block randomization with a block size of two using computer-generated random numbers. The participants received antihypertensive treatment with trichlormethiazide or without any thiazide diuretics for 12 weeks in a counterbalanced fashion (same patients received both therapies in a random order) as per an open-label, crossover design (see Fig. 3). A counterbalanced design was applied to the control participants for the order effect.

Considering the short half-lives of antihypertensive drugs, including trichlormethiazide, the washout period between the trials was set to 4 weeks. In addition, patients receiving trichlormethiazide or other thiazide diuretics were discontinued on those drugs and subjected to antihypertensive treatment without any thiazide drugs for at least 4 weeks before their participation. During their first visit and at every 4 weeks (namely at 0, 4, 8, and 12 weeks) for each 12-week trial (antihypertensive treatments without or with trichlormethiazide), their anthropometric profile and blood test reports were obtained. In addition, the participants were instructed to collect their urine over a full 24-h period at 4, 8, and 12 weeks of each trial and were trained to complete the HRQOL questionnaire at 12 weeks of each trial. The participants and CKD doctors were not blinded to group assignment considering that control of BP during the study period was extremely important, i.e., without knowing the allocation, a transient decrease or increase in BP was inevitable during 4 weeks before the first follow-up of each trial when transitioning between the trial phases of treatment with and without trichlormethiazide. In our short-term pilot study, we observed that variations in BP during the short period of 4 weeks might affect patient outcomes, including urinary volume, Uosm, and ADPKD-associated parameters. Therefore, a blinded study design was not approved by the ethics committee in the present study.

Regarding the selection of antihypertensive drugs, generally, a RAS inhibitor and/or a CCB was used as the antihypertensive drug. When trichlormethiazide was added to the treatments, dose reduction or discontinuation of RAS inhibitor or CCB was considered depending on BP management. Generally, trichlormethiazide therapy was initiated at the dose of 2 or 4 mg when the patient’s eGFR was ≥ 30 or < 30 mL/min/1.73 m^2^, respectively, with the target BP of 110/70 to 130/80. The prescriptions of antihypertensive drugs without or with trichlormethiazide could be adjusted by at least two of the three nephrologists (KU, CK, and YI) during the study period^37–39^. Moreover, when the patients’ BP became > 180/110 or < 100/60 mmHg, their participation in the trial was discontinued.

### Outcome measures

The primary study outcomes were the amount and osmolarity of the 24-h urine sample, whereas secondary outcomes were HRQOL, rate of decline in renal function, serum/urinary electrolytes, serum/urinary biomarkers associated with CKD and ADPKD progression, and office BP.

### Biochemical analyses

Urinary volume (mL) and Uosm (mOsm) were measured using 24-h urine samples collected at 4, 8, and 12 weeks of each trial. In daily clinical practice, salt intake of the patients was strictly controlled via a spot urine-guided method, which included informing patients of their spot urine-estimated salt intake at every visit to the outpatient clinic^40^ and performing periodical 24-h urine collections to accurately determine salt intake. Additionally, to assess the validity of the 24-h urine samples in the present trial, we ensured that the variation of 24-h urinary creatinine excretion was within 0.2 g/day and that none of the patients missed any urine sample collections. Uosm (mOsm) was also checked in fasting morning spot urine samples for sensitivity analysis, as previously described^41^. In addition, the established urinary biomarkers associated with CKD progression, namely, ACR (mg/gCr) and L-FABP level (μg/gCr) as well as urinary excretions of sodium and potassium (mEq/day), urea nitrogen (mmol/day), and osmolytes (mOsm/day) were evaluated using 24-h urine samples^14,18,42^. Additionally, urinary creatinine excretion (mg/day) was calculated to ascertain the accuracy of urinary collection.

Blood samples were collected in the morning in the fasting state every 4 weeks of each trial, and the slope of change in eGFR_Cr_ and eGFR_Cys_ values was calculated using a linear regression model. For these calculations, values at 4, 8, and 12 weeks but not at the baseline of each trial phase were used considering the acute hemodynamic effect of thiazide diuretics on eGFR. eGFR_Cr_ and eGFR_Cys_ were calculated using three-variable Japanese equations^43,44^. Serum sodium (mEq/L), potassium (mEq/L), and uric acid (mg/dL) levels were measured at 4, 8, and 12 weeks of each trial phase, whereas plasma biomarkers associated with ADPKD progression (plasma vasopressin [pg/mL] and copeptin [pg/mL]) were measured solely at 12 weeks of each trial. Vasopressin was measured via radioimmunoassay using the AVP kit (Yamasa, Tokyo, Japan). Copeptin was measured using a commercial enzyme-linked immunosorbent assay kit (WUHAN HUAMEI BIOTECH, Wuhan, China).

### HRQOL

HRQOL was measured using the KDQOL-SF Japanese version 1.3 with the subscales on general HRQOL (SF-36) and on QOL specific to kidney disease and dialysis (KDQOL) at every 12 weeks of each trial^45^. The physical component summary, mental component summary, and role/social component summary were calculated from the SF-36 scores, and the kidney disease component summary was calculated from the KDQOL scores, as described previously^46,47^.

### BP

Office BP was measured in the sitting position using an automated BP measuring device. With reference to the recommendations of the Japanese Society of Hypertension, measurements were performed twice after 10 min of rest, and mean BP was used for analysis^37^.

### Statistical analysis

Normal distribution of continuous data was tested using the Shapiro–Wilk test. Normally distributed data and skewed data are presented as mean ± standard deviation and median (interquartile range), respectively. Categorical data are expressed as *n* (%).

The paired Student’s *t* test or Wilcoxon signed-rank test was used for comparisons of continuous variables between both trials, and the unpaired *t* test or Mann–Whitney *U* test was performed for those between both groups divided by the sequence of antihypertensive treatment depending on the distribution of the parameters.

The mean values for variables, including primary outcomes (urinary volume and Uosm) and certain secondary outcomes (urinary ACR and L-FABP level; serum and urinary excretion of electrolytes; serum uric acid; urinary excretion of creatinine, urea nitrogen, and osmolytes; and blood pressure), measured at 4, 8, and 12 weeks of each trial phase, were calculated and used for comparison between the groups. For variables that were assessed only at 12 weeks of each trial phase, including the secondary outcomes of HRQOL, eGFR slope, and plasma vasopressin and copeptin levels, 12-week values were used for comparisons between the groups. Moreover, repeated-measured ANOVA was performed to detect significant differences in the primary outcomes between trials, within trials, and significant trials × time interactions for sensitivity analysis. Interim analyses were not performed.

Furthermore, subgroup analyses were performed to compare the effect of trichlormethiazide on urinary volume, Uosm, and eGFR slope in participants categorized according to the median duration of previous tolvaptan treatment, the change in antihypertensive agent dosage, and the change in RAS inhibitor dosage. To this end, analysis of covariance was performed using outcomes during the trial without trichlormethiazide as covariates and outcomes during the trial with trichlormethiazide as dependent variables.

With reference to the findings of a previous study that assessed Uosm in patients with ADPKD and a case report demonstrating the effect of a thiazide diuretic on polyuria induced by tolvaptan in patients with ADPKD, we showed that in 10 patients, Uosm increased by 78 mOsm with a standard deviation of 117 mOsm and a correlation between the trials of 0.8 (paired Student’s *t* test; β = 0.20; α = 0.05)^7,48^. Consequently, the statistical power was calculated as 0.84.

The SPSS software for Mac (ver. 25; IBM Corp., Armonk, NY, USA) was used to perform all statistical analyses. *P* < 0.05 indicated statistical significance.

## Supplementary Information

Supplementary Information 1.

## Data Availability

All data requests should be submitted to the corresponding author for consideration. Access to anonymized data may be granted after review.
