# Supplementary material for: The effect of trichlormethiazide in autosomal dominant polycystic kidney disease patients receiving tolvaptan: a randomized crossover controlled trial
Source: Sci Rep. 2021 Sep 3;11:17666. doi: 10.1038/s41598-021-97113-w (PMC8417075; doi:10.1038/s41598-021-97113-w)
Supplement: Supplementary file 1 — Supplementary Information 1. [file 41598_2021_97113_MOESM1_ESM.docx]

**The Effect of Trichlormethiazide in Autosomal Dominant Polycystic Kidney Disease Patients Receiving Tolvaptan: A Randomized Crossover Controlled Trial**

Kiyotaka Uchiyama*, MD, PhD,^1, 2^ Chigusa Kitayama, MD,^3^ Akane Yanai, MD,^4^ Yoshitaka Ishibashi, MD, PhD^2^

^1^Division of Endocrinology, Metabolism and Nephrology Department of Internal Medicine, Keio University School of Medicine, 35 Shinanomachi, Shinjuku-ku, Tokyo 160-8582, Japan

^2^Division of Nephrology, Japanese Red Cross Medical Center, 4-1-22 Hiroo, Shibuya-ku, Tokyo 150-8935, Japan

^3^Department of Nephrology, Japan Community Health Care Organization (JCHO) Sendai Hospital, Miyagi 981-8501, Japan

^4^Department of Nephrology, Tokyo Shinagawa Hospital, Tokyo 140-8522, Japan

Supplemental Material File Listing:

Table S1, Table S2, Table S3, and Table S4

Corresponding author:

Kiyotaka Uchiyama, MD, PhD

35 Shinanomachi, Shinjuku-ku, Tokyo 160-8582, Japan

Tel: +81-3-5363-3796; Fax: +81-3-3359-2745; E-mail: kiyo.0817.piyo@gmail.com

Table S1. Clinical and biochemical data of the first visit of each trial (antihypertensive treatments without or with trichlormethiazide)

| **Variables** | **Without trichlormethiazide (n = 10)** | **With trichlormethiazide (n = 10)** | ***P* value** |
| --- | --- | --- | --- |
| BMI (kg/m^2^) | 24.1 (23.2–27.6) | 24.2 (22.6–27.5) | 0.67 |
| GNRI | 111.8 ± 12.1 | 109.0 ± 11.5 | 0.07 |
| Systolic BP (mmHg) | 125.1 ± 14.5 | 129.7 ± 13.2 | 0.36 |
| Diastolic BP (mmHg) | 78.2 ± 10.2 | 83.9 ± 10.5 | 0.03 |
| Mean BP (mmHg) | 93.8 ± 10.3 | 99.2 ± 10.1 | 0.08 |
|  |  |  |  |
| **Kidney function** |  |  |  |
| eGFR_Cre_ (mL/min/1.73 m^2^) | 37.9 ± 21.8 | 40.0 ± 21.6 | 0.12 |
| eGFR_Cys_ (mL/min/1.73 m^2^) | 49.5 ± 33.7 | 50.5 ± 33.6 | 0.54 |
| UPCR (g/gCre) | 0.15 (0.10–0.28) | 0.10 (0.10–0.30) | 0.58 |
| Urinary osmolarity (mOsm) | 156.9 ± 57.6 | 130.6 ± 32.0 | 0.06 |
|  |  |  |  |
| **Serum biochemical analyses** |  |  |  |
| AST (IU/L) | 17.1 ± 3.0 | 15.2 ± 1.8 | 0.05 |
| ALT (IU/L) | 15.2 ± 5.8 | 12.3 ± 5.3 | 0.04 |
| γ -GTP (IU/L) | 20.0 (18.0–28.0) | 20.5 (16.3–31.3) | 0.8 |
| Sodium (mEq/L) | 140.9 ± 2.6 | 140.8 ± 2.7 | 0.84 |
| Potassium (mEq/L) | 4.47 ± 0.54 | 4.50 ± 0.66 | 0.77 |
| Chloride (mEq/L) | 105.5 ± 3.5 | 106.3 ± 3.8 | 0.29 |
| Albumin (g/L) | 4.14 ± 0.30 | 3.95 ± 0.22 | 0.04 |
| Fasting blood sugar (mg/dL) | 116.8 ± 25.4 | 111.2 ± 16.5 | 0.42 |
| LDL cholesterol (mg/dL) | 103.8 ± 22.5 | 119.0 ± 33.7 | 0.43 |
| HDL cholesterol (mg/dL | 54.7 ± 9.8 | 53.2 ± 9.0 | 0.24 |
| Triglyceride (mg/dL) | 105.7 ± 40.0 | 116.9 ± 33.9 | 0.19 |
| CRP (mg/L) | 0.13 ± 0.09 | 0.15 ± 0.15 | 0.64 |
| BNP (pg/mL) | 9.9 (9.1–22.7) | 17.6 (9.0–26.7) | 0.63 |
| Hemoglobin (g/dL) | 12.7 ± 1.3 | 12.4 ± 1.2 | 0.13 |
| Uric acid (mg/dL) | 6.28 ± 1.39 | 6.16 ± 1.23 | 0.69 |

Abbreviations: BMI, body mass index; GNRI, geriatric nutritional risk index; BP, blood pressure; CCVD, cerebrovascular/cardiovascular disease; eGFR, estimated glomerular filtration rate; Cr, creatinine; Cys, cystatin C; UPCR, urine protein-to-creatinine ratio; AST, aspartate aminotransferase; ALT, alanine aminotransferase; γ-GTP, γ-glutamyl transpeptidase; LDL, low-density lipoprotein; HDL, high-density lipoprotein; CRP, C-reactive protein; BNP, brain natriuretic peptide

Table S2. Details of tolvaptan dose and antihypertensive treatment during the study period

|  |  |  | **Without trichlormethiazide** | |  | **With trichlormethiazide** | |
| --- | --- | --- | --- | --- | --- | --- | --- |
| **Patient #** | **Tolvaptan (mg)** | **Group*** | **Baseline** | **Final** |  | **Baseline** | **Final** |
| 1 | 120 | 2 | Azilsartan 10 mg | Azilsartan 10 mg |  | Azilsartan 5 mg, trichlormethiazide 2 mg | Azilsartan 5 mg, trichlormethiazide 2 mg |
| 2 | 120 | 1 | Losartan potassium 50 mg, nifedipine 20 mg, atenolol 50 mg | Losartan potassium 50 mg, nifedipine 20 mg, atenolol 50 mg |  | Losartan potassium 50 mg, trichlormethiazide 4 mg, atenolol 50 mg | Losartan potassium 50 mg, trichlormethiazide 4 mg, atenolol 50 mg |
| 3 | 120 | 2 | Candesartan cilexetil 8 mg | Candesartan cilexetil 8 mg |  | Candesartan cilexetil 4 mg, trichlormethiazide 4mg | Candesartan cilexetil 4 mg, trichlormethiazide 2mg |
| 4 | 90 | 2 | Olmesartan medoxomil 20 mg, azelnidipine 16 mg, amlodipine5 mg | Olmesartan medoxomil 20 mg, azelnidipine 16 mg, nifedipine 60 mg |  | Olmesartan medoxomil 20 mg, azelnidipine 16 mg, trichlormethiazide 4 mg | Olmesartan medoxomil 20 mg, azelnidipine 16 mg, trichlormethiazide 4 mg |
| 5 | 120 | 1 | Telmisartan 40 mg | Telmisartan 40 mg |  | Telmisartan 20 mg, trichlormethiazide 2 mg | Telmisartan 20 mg, trichlormethiazide 2 mg |
| 6 | 120 | 1 | Nifedipine 20 mg | Nifedipine 40 mg |  | Nifedipine 40 mg, trichlormethiazide 4 mg | Nifedipine 40 mg, trichlormethiazide 4 mg |
| 7 | 60 | 1 | Losartan potassium 50 mg | Losartan potassium 50 mg |  | Losartan potassium 50 mg, trichlormethiazide 2 mg | Losartan potassium 50 mg, trichlormethiazide 2 mg |
| 8 | 90 | 2 | Olmesartan medoxomil 20 mg | Telmisartan 40 mg |  | Losartan potassium 50 mg, trichlormethiazide 4 mg | Losartan potassium 50 mg, trichlormethiazide 2 mg |
| 9 | 120 | 2 | Olmesartan medoxomil 40 mg | Olmesartan medoxomil 40 mg, nifedipine 20 mg |  | Olmesartan medoxomil 40 mg, trichlormethiazide 4 mg | Olmesartan medoxomil 20 mg, trichlormethiazide 4 mg |
| 10 | 60 | 1 | Losartan potassium 25 mg | Losartan potassium 25 mg |  | Losartan potassium 25 mg, trichlormethiazide 2 mg | Losartan potassium 25 mg, trichlormethiazide 1 mg |

*Group 1 was initiated on antihypertensive treatment with trichlormethiazide. Group 2 was initiated on antihypertensive treatment without trichlormethiazide.

Table S3. Subgroup analysis of the effect of trichloromethiazide on urinary volume and osmolarity

| **Variables** | **Urinary volume (mL/day)** | | |  | **Urinary osmolarity (mOsm)** | | |
| --- | --- | --- | --- | --- | --- | --- | --- |
|  | **Without trichloromethiazide** | **With trichloromethiazide** | ***P* for interaction** |  | **Without trichloromethiazide** | **With trichloromethiazide** | ***P* for interaction** |
| Duration of previous tolvaptan treatment |  |  | 0.93 |  |  |  | 0.98 |
| >43 months (n = 5) | 4459 ± 781 | 3493 ± 711 |  |  | 130.9 ± 26.7 | 174.3 ± 28.5 |  |
| <43 months (n = 5) | 4054 ± 722 | 3204 ± 457 |  |  | 152.1 ± 47.7 | 190.7 ± 26.5 |  |
|  |  |  |  |  |  |  |  |
| Change in antihypertensive agent dosage between the trial phases |  |  | 0.24 |  |  |  | 0.28 |
| Yes (n = 7) | 4156 ± 683 | 3337 ± 633 |  |  | 142.1 ± 44.2 | 178.7 ± 30.7 |  |
| No (n = 3) | 4491 ± 974 | 3378 ± 574 |  |  | 140.1 ± 25.6 | 191.2 ± 19.3 |  |
|  |  |  |  |  |  |  |  |
| Change in RAS inhibitor dosage between the trial phases |  |  | 0.93 |  |  |  | 0.29 |
| Yes (n = 5) | 3937 ± 688 | 3124 ± 576 |  |  | 145.8 ± 53.5 | 178.8 ± 37.6 |  |
| No (n = 5) | 4576 ± 710 | 3573 ± 557 |  |  | 137.3 ± 19.0 | 186.1 ± 15.4 |  |

Abbreviations: RAS, renin–angiotensin system.

Table S4. Subgroup analysis of the effect of trichloromethiazide on eGFR slopes

| **Variables** | **eGFR_Cre_ slope (mL/min/1.73m^2^/month)** | | |  | **eGFR_Cys_ slope (mL/min/1.73m^2^/month)** | | |
| --- | --- | --- | --- | --- | --- | --- | --- |
|  | **Without trichloromethiazide** | **With trichloromethiazide** | ***P* for interaction** |  | **Without trichloromethiazide** | **With trichloromethiazide** | ***P* for interaction** |
| Duration of previous tolvaptan treatment |  |  | 0.62 |  |  |  | 0.58 |
| >43 months (n = 5) | −0.67 ± 2.52 | 0.77 ± 1.30 |  |  | −0.06 ± 4.31 | 0.39 ± 1.81 |  |
| <43 months (n = 5) | 0.08 ± 1.02 | 0.69 ± 1.82 |  |  | −0.17 ± 1.13 | 0.98 ± 2.11 |  |
|  |  |  |  |  |  |  |  |
| Change in antihypertensive agent dosage between the trial phases |  |  | 0.13 |  |  |  | 0.46 |
| Yes (n = 7) | 0.33 ± 1.12 | 0.13 ± 1.01 |  |  | 0.05 ± 1.21 | 0.85 ± 2.54 |  |
| No (n = 3) | −1.75 ± 2.68 | 2.12 ± 1.67 |  |  | −2.37 ± 3.07 | 2.16 ± 2.61 |  |
|  |  |  |  |  |  |  |  |
| Change in RAS inhibitor dosage between the trial phases |  |  | 0.52 |  |  |  | 0.59 |
| Yes (n = 5) | 0.57 ± 1.27 | 0.15 ± 1.11 |  |  | 1.73 ± 2.44 | −0.35 ± 1.13 |  |
| No (n = 5) | −1.16 ± 2.07 | 1.30 ± 1.72 |  |  | −1.96 ± 2.32 | 1.71 ± 2.01 |  |

Abbreviations: eGFR, estimated glomerular filtration rate; Cre, creatinine; Cys, cystatin C; RAS, renin–angiotensin system.
